# Secure Key Establishment Mechanism for Smart Sensing System Based Robots Network

**DOI:** 10.3390/s20071970

**Published:** 2020-04-01

**Authors:** Qi Xiao, Yunchuan Qin, Cheng Xu, Kenli Li

**Affiliations:** 1 College of Computer Science and Electronic Engineering, Hunan University, Changsha 410000, China; xiaoqi.0909@163.com (Q.X.);; 2 SZZT Electronics Co., Ltd., SZZT Industrial Park, No.3 Tongguan Rd, Guangming District, Shenzhen 518000, China

**Keywords:** multi-robot systems, key establishment, high-order polynomial, smart robot

## Abstract

The smart robot is playing an increasingly important role in the social economy, and multi-robot systems will be an important development in robotics. With smart sensing systems, the communications between sensors, actuators, and edge computing systems and robots are prone to be attacked due to the highly dynamic and distributed environment. Since smart robots are often distributed in open environments, as well as due to their limited hardware resources and security protection capabilities, the security requirements of their keys cannot be met with traditional key distribution algorithms. In this paper, we propose a new mechanism of key establishment based on high-order polynomials to ensure the safe key generation and key distribution. Experiments show that the key establishment mechanism proposed in this paper guarantees the security of keys; its storage cost and communication cost are smaller than state-of-the-art mechanisms; and it allows robot components to join and leave the network dynamically, which is more suitable for multi-robot systems.

## 1. Introduction

With the fierce competition in the global market and the gradual increase in labor costs, as well as the booming of big data, cloud computing, Internet of Things [1], and artificial intelligence, the robotic industry has become increasingly important. Due to the limitations of its own performance, a single robot cannot successfully complete many tasks, and it must rely on multiple robots to work together. Multi-robots are widely used in real life, such as explosion protection, disaster relief, industrial production, and distribution logistics. The emergence of multi-robot systems is also considered to be one of the top ten robotic technology challenges that will have a major impact on the social economy in the next 5–10 years [2].

With the increasing application of multi-robot systems, there have been many studies in this field, including the study of robot motion trajectories [3], the cooperation of multi-robots [4], and robot safety research. This article focuses on the information security of multi-robot systems. In smart sensing systems, the sensors and the robots need to communicate with each other when performing a task, so that they can complete the task more accurately, efficiently, and intelligently. For some special tasks, such as military operations, personal information collection, etc., robots must maintain the confidentiality of tasks and the security of information [5]. The China Software Testing Center CCID Robot Testing and Certification Center released a report analyzing the information security issues of mainstream public service robots in China. The report shows that service robots are close to the public life, and the information security vulnerabilities may lead to leaks, which may cause harm to people in serious cases. Therefore, the safe operation of multi-robot systems requires that the communication among sensors, actuators, and edge computing systems in the system be safe and reliable, and the security of multi-robot networks receives more and more attention from researchers.

Cryptographic algorithms are the important means to solve the network security problems. The most critical factor is the security of keys. In multi-node networks, the research on key security has gained significant development. Zhang [6] provided a practical design guide for a secure key generation system that can be changed by a different design. Thai [7] proposed a group key generation mechanism in the physical layer to deliver information through multiple antennas in a wireless network topology. Eschenauer [8] proposed a key pre-distribution algorithm, which performs key pre-distribution by establishing a key pool, but this key pre-distribution algorithm cannot guarantee 100% interworking between nodes. Sencun [9] proposed a localized encryption and authentication protocol (LEAP) to solve the problem of different security requirements when exchanging different types of data between nodes. However, after the initial deployment of the node, the interworking issues when the node and key changes cannot be solved. Jiyong [10] proposed a time-based key management protocol, but the protocol has connectivity issues for node deployment between different time slots. Naor [11] proposed a description schemes for distributing between n servers the evaluation of a function f, which can be used to distribute the operation of a KDC. However, this schemes cannot identify the attacked node in time, and it cannot update the key in time when a component being attacked appears, to avoid the information leakage.

Currently, there is much research on key pre-distribution schemes in wireless sensor networks, and some of them can also be applied to smart robots networks. The studies include those on the Blom-Blundo et al. key predistribution schemes and new generalized Blom-Blundo et al. key predistribution schemes [12], secure and energy-efficient traffic-aware key management scheme (EETKM) [13], sequence-based encryption Key management scheme (SKM) [14], and authentication key establish protocol [15], among others. However, the components in the multi-robot network have the characteristics of limited resources, more flexibility, mobility, vulnerability to be attacked, etc. Thus, we consider a simpler and more suitable key management scheme for the multi-robot network.

Most studies about the security of wireless sensor networks focus on the security of communication between nodes. Due to the complexity and dynamic mobility of the multi-robot system, it is vulnerable to physical attacks such as cold boot attack. Attackers can further launch attacks such as Sybil attack through the attacked robots, thus making the multi-robot network face the serious security threats. In safety-critical scenarios, traditional sensor network security mechanisms cannot provide adequate security protection for multi-robot systems. Multi-robots are highly dynamic and distributed, e.g. nodes join and exit the network frequently, making it difficult for the traditional key distribution and key authentication maintain the key security. Thus, a more suitable mechanism should be proposed for the safety-critical multi-robot system.

Considering to the high dynamics of robots, the limited hardware resources, and different security protection capabilities in multi-robot networks, this paper proposes a security key establishment mechanism based on high-order polynomial. The main contributions of this paper are summarized as follows:

1. This paper proposes a new and secure key establishment mechanism, in which keys are generated in the key center in the cloud and sent to the communication components through the key update instruction, which contains high-order polynomial coefficients. The attacked components list included in the instruction are broadcast to all components.
2. The key is generated in the key center and distributed periodically, which allows the communication components to join and exit the network at any time. The communication component only needs a simple calculation to get the key, which is resource-saving. The attacked components list would be broadcast to all components, preventing the attacked components from disrupting the communication in the network.
3. Comparing with other mechanisms, the mechanism proposed in this paper has high security, high dynamics, and low resource consumption, which is more suitable for multi-robot systems.

## 2. Related Work

The multi-robot system refers to acquiring its own state information and surrounding environmental information through a variety of different sensors; merging the acquired information; coordinating the behaviors of multiple robots in combination with the tasks assigned by the system; and cooperating with each other to finally complete the task. The research of multi-robot began in the 1980s, mainly focusing on multi-robot architecture, collaborative communication, motion modeling, and path planning [16].

Key establishment refers to the process of generating an available, shared secret key between one or more entities [17]. Key establishment includes key generation and negotiation, key transmission, etc. Among them, key generation and negotiation refers to a process of establishing a shared secret key between entities, wherein any entity cannot predetermine the value of the key.

Classical cryptographic algorithms are widely used, and their security has been verified, but, with the advancement of technology, the probability of these cryptographic algorithms relying on computing power to ensure security begins to increase. For example, the RSA algorithm has high security due to the difficulty of factoring. With the advent of the SHOR algorithm and the deepening of quantum computer research, the parallelism of quantum computing can be used to quickly resolve large numbers of prime factors. Therefore, the introduction of quantum computing is likely to be able to crack the widely used RSA public key encryption system. The emergence of quantum cryptography algorithms has broken the limitations brought by hash algorithms based on computing power [18]. Therefore, for different applications or systems, more secure and efficient algorithms should be proposed.

The AES algorithm is used in this paper to compute the keys, and it is a symmetric cryptographic algorithm. It has the advantages of strong security, high flexibility, high performance, and high efficiency. It is one of the block cipher algorithms [19]. The block cipher algorithm divides the input data into fixed-length packets. After the encryption is completed, the ciphertext output by each packet has the same length as the packet length of the input plaintext. $N_{r}$ indicates the number of rounds of encryption of a data packet (the number of encryption rounds is related to the length of the key). Each round of encryption requires an extended key whose length is consistent with the length of the input data packet. The encryption key externally input into the encryption algorithm has a limited length. Therefore, in the AES algorithm encryption process, the key extension program length-expands the external key to obtain a longer bit string to generate each round of encryption key and decryption key.

## 3. Security Key Establishment Mechanism Based on the High-Order Polynomial

### 3.1. Overall Design

This paper proposes a security key establishment mechanism based on the high-order polynomial. It discusses the key management among the multiple smart components (such as sensors and actuators) on a single robot in the multi-robots network, and it is based on a symmetric key distribution algorithm. The key-center (or the cloud) sends the key in a time slot to the components by sending the coefficients of a polynomial. The components restore the coefficients to a polynomial and plug the private information themselves into the polynomial to obtain the key. The components are divided into multiple communication groups (or clusters) in the typical bus topological structure, according to the attribution of the components. The keys in a cluster are generated and distributed by the key center periodically.

**Figure 1:**
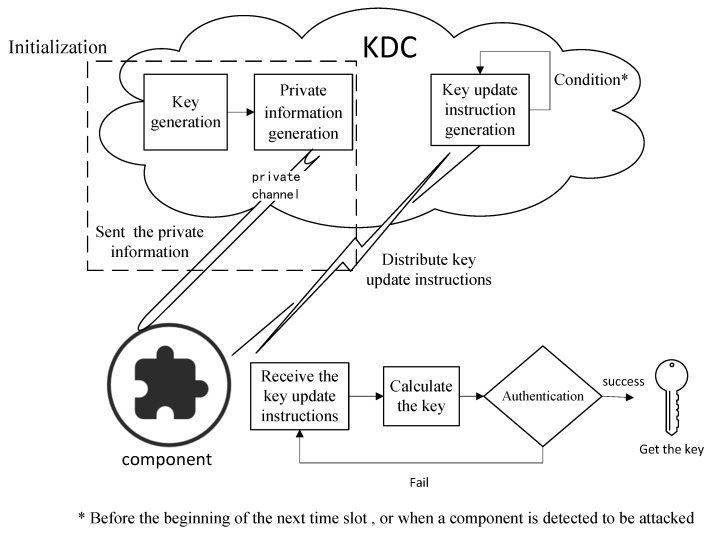
The process of the security key establishment mechanism.

At the beginning of a period, all keys in this period are generated by the key center and distributed to the components before each time slot begins. The process is presented as follows, and it is shown in Figure 1.

1. At the beginning, the key center calculates the communication session key ($K_{1,CID},K_{2,CID},\ldots ,$$K_{N,CID}$) of a cluster in the N time slots, based on the cluster identification information CID, and saves them in the key center.
2. The key center generates private information for each communication component in the cluster. The private information is sent to each component through a secure private channel at initialization. It is privately stored by each component, and is used to calculate the keys in the next few time slots.
3. Before the beginning of the next time slot *i*, or when a component is detected to be attacked, the key center calculates the coefficients of a set of polynomials and generates a key update instruction, which is broadcast to components of a cluster.
4. The key center broadcasts the ID information of the components which are attacked to all components in the key update instruction.
5. After receiving the key update instruction, the component restores the polynomial and calculates the key according to the formula.
6. After the calculation, the component uses the message authentication code to identify whether the received information is true and complete.
7. The component updates the key based on the authentication result.

### 3.2. Key Factor Initialization

On a smart robot, the communication components (such as sensors) communicate and cooperate closely with each other. Some other smart robots that have business relevance to the robot also communicate frequently with it. Therefore, all communication components on the smart robot form a communication group called cluster [20]. In each cluster, a component that can communicate with all other components in the cluster is called the cluster head component [21]. In general, the cluster head communicates with other components in the cluster using one or more full-duplex, half-duplex communication links such as UART, Ethernet, SPI, I2C, and CAN, and each cluster head communicates with cloud based on the IP network, such as GPRS, LTE, NBloT, LowPAN, etc. Therefore, to prevent the attackers from intercepting intra-cluster communication information through a shared medium or attacking the node itself, each component in the cluster needs to hold a common session key to encrypt and decrypt the communication information.

The communication session key between the intra-cluster communication components is distributed by the Key Distribution Center (KDC) located in the cloud, which greatly reduces computing resources for communication components with weak computing power. Each communication component holds a private and unique information identification value NID, and each cluster has a cluster identification CID. Before the start of communication for one cycle, the KDC generates a communication session key group $\left\{K_{1,CID},K_{2,CID},\ldots ,K_{N,CID}\right\}$ for each normal communication component within the cluster CID, in the initial stage, the secret value $S_{NID}$ required to calculate the session key is delivered to each communication component through the private channel.

In the initial stage, the key center uses the AES (Advanced Encryption Standard) algorithm to generate the communication session key of the components in the cluster. The AES algorithm is a highly efficient and high-security symmetric encryption algorithm. The algorithm encrypts the data with low computational cost and high security. Since the data encrypted by the key center do not need to be decrypted, the key center can save the key to ensure the privacy of the information.

Before the key generation begins, the key center first generates a random number *R* through a reliable random source, which is stored as a root key by the key center and protects its privacy. For the key of the component within the cluster CID in the *i* time slot, the key center is generated as shown in Equation (1).

(1) $$K_{i,CID}=AES\left(AES\left(R,CID\right),i\right)$$

where *i* is the time slot number and CID is the unique identification number of the cluster.

The formula is used to generate the key of the component in the cluster, including the flag information of the cluster. The uniqueness of the flag information ensures the uniqueness of the key. Since only the private value of the generated key is stored in the key center, *R*, the storage cost of the key information is reduced, and the security of the key is greatly enhanced.

**Definition** **1.** *The private information of the component NID is $S_{NID}$, which is a ciphertext calculated by the AES algorithm by the key center according to the node ID of each component and the random number R generated by the key center. The generation of private information of the component NID is shown in Equation (2).*

(2) $$S_{NID}=AES\left(R,NID\right)$$

In the key factor initialization phase, the private information held by each component $S_{NID}$ is written by the key center into the corresponding component through the protected channel according to its identification information NID in a controlled environment. Each component saves and protects its privacy in *N* time slots. This private information is used by each component for key calculations in subsequent *N* time slots.

### 3.3. Key Update Algorithm

#### 3.3.1. Key Update Instruction

To improve the security of the key of communication session between components, the normal components in the cluster can respond to the attack in time. The key center will distribute the key update instruction to all the components in the cluster before the start of each time slot or when any component is detected to be attacked. The key update instruction sent by the key center to the components mainly carries the coefficient information of the key update polynomial $F_{i}\left(X\right)$, the list of attacked components, and the list of random numbers. The key update polynomial is composed by the cluster key $K_{i,CID}$, the interference polynomial δ(X), and the identification information of each component. The formula is shown in Equation (3).

(3) $$F_{i}\left(X\right)=AES\left(S_{NID},i\right)+K_{i,CID}*\delta \left(X\right)=a_{n-1}X^{n-1}+a_{n-2}X^{n-2}+\ldots +a_{1}X+a_{0}$$

where $S_{NID}$ is the private information held by each component, *i* is the time slot value, and $\left\{a_{0},a_{1},\ldots ,a_{n-1}\right\}$ is the polynomial coefficient. When the number of normal components in the cluster *m* is greater than the number of components being attacked, then n=m+2; otherwise, n=p+3.

In the key updating, the key center introduces an interference polynomial δ(X) in order to prevent the attacked components from getting the communication session key of the next time slot and excluding the interference of attacked components to the intra-cluster communication. The δ(X) is defined as Equation (4). The coefficients of δ(X) consist of the identification information of the attacked components and some random numbers. The interference polynomial δ(X) lists the identification information of the attacked components, so that the attacked components would not be able to obtain the key of the new time slot, even though they relive the key update instruction. When the number of attacked components *p* is less than the number of normal components *m*, the introduction of the random number list makes the highest index of the key update polynomial achieve the number m+1. When the number of attacked components *p* is greater than the number of normal components *m*, the equation only introduces two random numbers $b_{1}$ and $b_{2}$, and the highest index of the key update polynomial is p+2.

(4) $$\delta \left(X\right)=\left(X-ID_{1}\right)\left(X-ID_{2}\right)\ldots \left(X-ID_{p}\right)\left(X-b_{1}\right)\left(X-b_{2}\right)\ldots \left(X-b_{k}\right)$$

where $ID_{1},ID_{2},\ldots ,ID_{p}$ is the ID of the attacked components, *p* is the number of attacked components, and $b_{1},b_{2},\ldots ,b_{k}$ is a random number generated by a key source random source. When p<m, p+k=m+1; otherwise, k=2.

Equation (3) shows that the key update polynomial is determined by the coefficients $\left\{a_{0},a_{1},\ldots ,a_{n-1}\right\}$, and the index of the polynomial coefficient is *n*. The coefficient calculation of the key update polynomial is performed in the key center. When the number of normal components (*m*) in the cluster is greater than the number of attacked components (*p*), the IDs of all normal components in the cluster, the private information value corresponding to the component, and the anti-attack pairs $\left(T_{1},S_{T_{1}}\right)$ and $\left(T_{2},S_{T_{2}}\right)$, which are generated randomly, are brought into Equation (3) to get Equation (5). In the calculation of polynomial coefficient, the addition of random number pairs makes it impossible for the attacker to obtain the private information of all components, and the key of the next time slot cannot be calculated due to the existence of the random number, thereby improving the security of the key.

(5) $$\left\{\begin{array}{c} AES\left(S_{ID_{1}},i\right)+K_{i,CID}*\delta \left(ID_{1}\right)=a_{n-1}ID_{1}^{n-1}+a_{n-2}ID_{1}^{n-2}+\ldots +a_{1}ID_{1}+a_{0}\\ AES\left(S_{ID_{2}},i\right)+K_{i,CID}*\delta \left(ID_{2}\right)=a_{n-1}ID_{2}^{n-1}+a_{n-2}ID_{2}^{n-2}+\ldots +a_{1}ID_{2}+a_{0}\\ \ldots \\ AES\left(S_{ID_{n}},i\right)+K_{i,CID}*\delta \left(ID_{n}\right)=a_{n-1}ID_{n}^{n-1}+a_{n-2}ID_{n}^{n-2}+\ldots +a_{1}ID_{n}+a_{0}\\ AES\left(S_{T_{1}},i\right)+K_{i,CID}*\delta \left(T_{1}\right)=a_{n-1}T_{1}^{n-1}+a_{n-2}T_{1}^{n-2}+\ldots +a_{1}T_{1}+a_{0}\\ AES\left(S_{T_{2}},i\right)+K_{i,CID}*\delta \left(T_{2}\right)=a_{n-1}T_{2}^{n-1}+a_{n-2}T_{2}^{n-2}+\ldots +a_{1}T_{2}+a_{0} \end{array}\right.$$

When the number of normal components (*m*) in the cluster is less than the number of the attacked components (*p*), the key center will automatically generate *x* pairs of anti-attack number $\left(X_{j},S_{X_{j}}\right)$, so that m+x=p+2. The key center can bring the ID of all normal components and their corresponding private information values, together with the *x* pairs of anti-attack number randomly generated above, into Equation (3), obtaining the equation set shown in Equation (6).

Solving the equation set in a finite field, a set of coefficient vectors $\left[a_{0},a_{1},\ldots ,a_{n-1}\right]$ can be obtained. The key center composes the coefficient vectors together with the identification information list of the attacked components and the random number list in the interference polynomial δ(X) to form a key update instruction, and then sends it to all communication components in the cluster by broadcast. It should be noted that the attacked components do not receive the key update instruction.

(6) $$\left\{\begin{array}{c} AES\left(S_{ID_{1}},i\right)+K_{i,CID}*\delta \left(ID_{1}\right)=a_{n-1}ID_{1}^{n-1}+a_{n-2}ID_{1}^{n-2}+\ldots +a_{1}ID_{1}+a_{0}\\ AES\left(S_{ID_{2}},i\right)+K_{i,CID}*\delta \left(ID_{2}\right)=a_{n-1}ID_{2}^{n-1}+a_{n-2}ID_{2}^{n-2}+\ldots +a_{1}ID_{2}+a_{0}\\ \ldots \\ AES\left(S_{ID_{n}},i\right)+K_{i,CID}*\delta \left(ID_{n}\right)=a_{n-1}ID_{n}^{n-1}+a_{n-2}ID_{n}^{n-2}+\ldots +a_{1}ID_{n}+a_{0}\\ AES\left(S_{X_{1}},i\right)+K_{i,CID}*\delta \left(X_{1}\right)=a_{n-1}X_{1}^{n-1}+a_{n-2}X_{1}^{n-2}+\ldots +a_{1}X_{1}+a_{0}\\ \ldots \\ AES\left(S_{X_{x}},i\right)+K_{i,CID}*\delta \left(X_{x}\right)=a_{n-1}X_{x}^{n-1}+a_{n-2}X_{x}^{n-2}+\ldots +a_{1}X_{x}+a_{0} \end{array}\right.$$

#### 3.3.2. Handling of Key Update Instructions

The process of a component receiving the key update instruction is shown in Algorithm 1. After receiving the key update instruction sent by the key center, the component takes out the coefficient information $\left[a_{0},a_{1},\ldots ,a_{n-1}\right]$, which is carried in the instruction, to construct the key distribution polynomial $F_{i}\left(X\right)=a_{n-1}X^{n-1}+a_{n-2}X^{n-2}+\ldots +a_{1}X+a_{0}$. The ID list of the attacked components and the random number list which are published by the key center can construct the polynomial $\delta \left(X\right)=\left(X-ID_{1}\right)\left(X-ID_{2}\right)\ldots \left(X-ID_{p}\right)\left(X-b_{1}\right)\left(X-b_{2}\right)\ldots \left(X-b_{k}\right)$. According to the formula shown in Equation (7), we can calculate the key $C_{i,CID}$, which is the session key of cluster CID in the next time slot.

(7) $$C_{i,CID}=\left(F_{i}\left(X\right)-AES\left(S_{NID},i\right)\right)/\delta \left(X\right)=\left(F_{i}\left(NID\right)-AES\left(S_{NID},i\right)\right)/\delta \left(NID\right)$$

where *i* is the time slot number.

Each component takes its own node identifier NID, and the private information $S_{NID}$ saved by the component itself, into Equation (7) to obtain the intra-cluster communication session key $C_{i,CID}$ in the next slot. Since the attacked components have already been published by the key center, even though their own identification information and private information value are brought into Equation (7), they also cannot calculate the key value in the next time slot, due to the interference polynomial δ(NID)=0.
**Algorithm 1** The processing algorithm of key update instruction.**Input:**$\left\{a_{0},a_{1},,,,,a_{n-1}\right\}$, Coefficient value in the key update instruction distributed by the key center. $\left\{ID_{1},ID_{2},\ldots ,ID_{p},b_{1},b_{2},\ldots ,b_{k}\right\}$, List of attacked component ID numbers and random numbers delivered by the key center.**Output:** The new key $C_{i,CID}$ of the Time Slot *i*1:// assault polynomial δ(X)2:$Generate_{a}ssault\left(x\right)\leftarrow \left(x-ID_{1}\right)*\left(x-ID_{2}\right)\ldots *\left(x-ID_{p}\right)*\left(x-b_{1}\right)*\left(x-b_{2}\right)\ldots *\left(x-b_{k}\right)$;3://Constructing key issuing polynomial4:$F_{i}\left(X\right)=a_{n-1}X^{n-1}+a_{nk-2}X^{n-2}+\ldots +a_{1}X+a_{0};$5://ring the component ID number NID into the issuing polynomial6:$F_{i}\left(NID\right)=a_{n-1}NID^{n-1}+a_{n-2}NID^{n-2}+\ldots +a_{1}*NID+a_{0};$7://Calculate the new key value using Equation (7)8:**if**$Generate_{a}ssault\left(NID\right)==0$**then return** false;9:**else**10:  $C_{i,CID}=\left(F_{i}\left(NID\right)-AES\left(C_{NID},i\right)\right)/Generate_{a}ssault\left(NID\right);$ **return** $C_{i,CID}$11:**end if**

### 3.4. Message Authentication and Authentication

To identify the key value calculated above, whether it is distributed from the key center, and whether it has been tampered, the key update instruction carries the information for verification when it is sent by the key center to the components—the message authentication code (MAC). After receiving the key update instruction, each component takes out its last byte as the MAC. After the key is calculated, the MAC is used to verify the authenticity and integrity of the key.

After the key center generates the key update instruction, the message authentication code is added at the end of the instruction. The process is as follows:

1. Assuming that the key update instruction to be sent is the plain text KM, the key center inputs the intra-cluster communication key of the next time slot ($C_{i,CID}$) and the plain text into the HMAC algorithm to obtain a message authentication code KC=H(KM).
2. The message authentication code KC is attached to the original message KM to generate a new plain text $KM^{{}^{\prime }}=\left[KM\cup KC\right]$.
3. The new plain text $KM^{\prime }$ is distributed as a key update instruction to the communication components.

When a component in the cluster receives the key update instruction, it calculates the character length of the instruction, for example *N*, and performs as following:

1. Extract the first N-16 bytes in the instruction to get the key update information instruction NM, and extract the last 16 bytes of the instruction as the check code MC which is sent by the key center.
2. Use the key update algorithm mentioned in the previous section to calculate the intra-cluster communication session key as $NK_{i,CID}$.
3. Input the communication session key $NK_{i,CID}$ and the key update information instruction NM into the HMAC algorithm to obtain a message authentication code NC=H(NM).
4. Compare the message authentication code NC with the instruction’s check code MC. If NC matches with MC, it means that the key which the component received is correct and it comes from the key center, and the key $NK_{i,CID}$ becomes the new session key, which replaces the key of last time slot. If there is no match, the component notifies the key center to re-distribute the key update instruction.

### 3.5. Key Update

When a communication component is attacked, the key information and private information stored in this communication component is cleared automatically. When the key center communicates with the communication component, if the key center cannot receive the response from the communication component three times, or the message responded from the communication component is not encrypted using the communication session key of the current time slot, the component is considered to have been attacked or has something wrong. Then, the key center adds the ID of this communication component to the attacked list and broadcasts it to all communication components in the cluster. At the same time, the communication key in this time slot is discarded, and the key center initiates the key update process.

When a new communication component is added to a cluster, the cluster head reports the ID of the new component to the key center, and the key center adds the ID of the new component to the normal components list. Before the start of the next time slot, the key center generates a private information value $S_{NID}=AES\left(R,NID\right)$ for the new component, and delivers the private information to the component through the private channel. When constructing the key update instruction of next time slot, the key center adds the information of the new component into the calculation, so that the new component can receive the intra-cluster communication session key in the next time slot.

### 3.6. Key Validity

In the key generation and distribution algorithm proposed in this paper, the key center only generates and distributes a group of keys in a period; the group contains the keys of the next *N* time slots. This group of keys is valid only in a period, and the key corresponding to each time slot is valid only in that time slot. The key is invalid outside the period or outside the corresponding time slot. The continuous update of the key can guarantee the security of the session to a greater extent because, the longer the key is used, the greater the chance of it being stolen, and the greater the risk of information leakage. Once a key is compromised, the longer is the key’s validity period, the greater is the loss. The longer a key is used, the easier it is for an attacker to perform cryptanalysis on multiple ciphertexts encrypted with the same key, and the longer the attack time is left for the attacker.

The validity period setting of the key is related to the parameters of the system. This paper mainly analyzes the three parameters that affect the validity period of the key: (1) The number of communication components. The larger is the size of the communication components, the more resources are consumed by the key distribution, the higher are the security requirements of the key, and the validity period of the key should achieve a balanced value. (2) The system security level. The higher is the security level of the system, the shorter the key validity period should be set, so that the attacker does not have enough time to analyze the key. (3) The network communication capability in system. If a system has good network communication capability, the communication key validity period can be shortened to ensure the security of the key. If the network communication capability is not too strong in a system, the key validity period should be extended to alleviate the communication pressure of the system. Therefore, the key validity period can be set according to the configuration parameters of the system and the practical applications.

### 3.7. Performance Analysis

The key generation and distribution algorithm proposed in this paper has the characteristics of high security, small overhead, and strong scalability. Its performance can be analyzed from the aspects of security, effectiveness, and flexibility.

#### 3.7.1. Security

(1) Key security in component. When an attacker attacks a communication component in the system, the key related information in the component is protected by the component privately and it is difficult for the attacker to obtain. Even if the attacker gets the useful key related information, the key center will broadcast the ID of the attacked components to all relevant components after detecting the abnormality of the component, and start a key update process to distribute the polynomial coefficients about new key to all relevant components. The new key update instruction lists the component above as the attacked component, so that the attacked component cannot calculate the new key of the next time slot. Therefore, after the key update instruction is distributed, all the information sent by the attacked component to the communication bus will not be recognized by other components due to the wrong key, and it will not be able to decrypt all the information in the cluster, making the attacker unable to steal useful information relying on the current information, or to interfere with the communication of other components in the cluster, ensuring that, even if some communication components in the system are attacked, the communication among the other components in the cluster can still perform normally.

(2) Forward and backward security. According to the key generation algorithm, we can find that the key center holds the master key *R*, and no communication component stores the related information about master key. Therefore, even if an attacker attacks some communication components in the cluster, he only can get the key of current time slot. The key of next time slot cannot be calculated without the master key *R*, and the information encrypted by the new key cannot be decrypted by attacker. Therefore, the key distribution algorithm proposed in this paper has forward security. Due to the security of the AES algorithm, even if the attacker has obtained the key of this time slot, the attacker cannot break through the AES encryption algorithm to get all the keys before the current time slot. Before the new time slot starts, the component would destroy the key of previous time slot; thus, there are no previous keys stored in the component, and the attacker cannot get all keys before current time slot, which means the key distribution algorithm proposed in this paper has backward security.

(3) Anti-collusion. If attackers successfully break some components in the system, they can conspire and cooperate with each other, and then calculate all the keys, which may eventually break the entire network. Therefore, a perfect key distribution mechanism should be able to resist the collusion between the newly joined node and the attacked node. In our key distribution algorithm, even if the attackers get the private information of some components and know the communication key of the current time slot, there is also no way to calculate the private information of the remaining components, and, after new key update instructions are distributed, they cannot participate in communication within the cluster. Even if the attacker knows the ID and private information of all components, they cannot break the polynomial because they cannot get the random number generated by the key center, and cannot calculate the key for the next time slot.

(4) Invulnerability. When a communication component in the system is attacked, the key center responds immediately, broadcasts the ID of the attacked component to all components in the cluster, and redistributes a new key update instruction to all normal components, to exclude the attacked component quickly. Attackers cannot affect the communication of other components by attacking one or more components; the key distribution algorithm proposed in this paper is high invulnerability.

#### 3.7.2. Effectiveness

(1) Storage cost. The memory space of each communication component on the smart robot in the multi-robots network is limited. When designing the key distribution algorithm, we consider this feature of the component fully in this paper, and only the key related information $S_{NID}$ is required to store in the component, superadd the ID of itself and time slot number *i*, thus the memory usage of the component is very low.

(2) Quantity of information. After the key is generated, the key center sends the key related information $S_{NID}$ to each communication component through the private channel, which has less information and does not occupy the normal communication network bandwidth. Before each time slot starts, the key center would distribute key update instructions to each component. The instruction mainly contains *m* (*m* is the number of normal components in the cluster) dimension coefficient vectors $\left[a_{0},a_{1},\ldots ,a_{m-1}\right]$, the ID list of *p* (*p* is the number of attacked components) attacked components, and a one-byte message authentication code.

(3) Energy consumption. The key generation is performed in the key center, and the calculated quantity is negligible compared with the massive storage and large calculation support in the cloud. For the communication component, the calculated quantity of a component to calculate a key is small, and the storage cost is small, so that the configuration requirements of the component are low. During the key distribution process, fewer bytes are transmitted in the key update instructions, which reduces The burden of communication.

#### 3.7.3. Flexibility

The key distribution mechanism proposed in this paper is flexible enough to adapt to various application scenarios in a multi-robot network. It is mainly reflected in the following aspects.

(1) Transferability. In a multi-robot network, many smart robots need to move. In our key distribution algorithm, the key center in the cloud communicates with the components through the network. No matter where the smart robot moves, the key update can perform as normal, as long as the network is still connected to the cloud.

(2) Scalability. Because most work of the key distribution algorithm proposed in this paper is in the cloud, the scale of nodes that it can handle is very large. When the communication components need to be added in the system or deleted, the key center obtains the ID of the added/deleted components. When distributing the key update instruction, the key center recalculates the key update polynomial coefficient based on the current active nodes.

## 4. Experiments and Result Analysis

The key distribution algorithm based on high-order polynomial proposed in this paper has great advantages in terms of storage cost and communication cost compared with other algorithms. The cloud-based key center also provides powerful support for the processing of massive communication components. In the simulation experiment of our algorithm, the key center is built on the OpenStack-based cloud host cluster. The communication components involved in communication are composed of intelligent robot readers, PIN keyboards, cameras, and other components. The communication links between the components are one or more in full-duplex, half-duplex communication links such as UART, Ethernet, SPI, I2C, CAN, etc.

Table 1 compares the security key establishment mechanism based on high-order polynomial with the existing schemes in terms of storage cost, communication cost, revocation capability, collusion resistance, and robustness. This section compares several key distribution algorithms, including the component exemption time-limited group key distribution scheme [22], the anti-collusion key distribution scheme with revocation ability [23], the limited self-healing key distribution scheme named LiSH [24], and its enhancement scheme Lish+ [20]. Although the solution proposed by Jiang [22] is better in terms of storage cost and communication cost, its revocation capability is limited, and the user cannot be revoked by the key center until the end of its life cycle. In addition, this scheme is also unable to resist collusion attacks. The scheme proposed by Du [23] and Biming [25] can only partially resist collusion attacks. In the following, we perform a detailed performance evaluation of the security key establishment mechanism based on high-order polynomial coefficients proposed in this paper from the perspective of storage and communication.

### 4.1. Storage Cost

In the security key establishment mechanism based on high-order polynomial proposed in this paper, a communication component can store some key related information in a key distribution period, including the component’s ID and the key private information $S_{ID}$ received from the key center. During the entire key distribution period, the component only needs to store these two values to calculate the key of next time slot. After the key update instruction is distributed, the communication component fetches the data in the instruction, calculates the key of the next time slot, and keeps it in the component. Then, it deletes the key of the previous time slot. Therefore, during the entire key distribution process, the communication component only needs to store the component identification ID (4 bytes) and the component private information $S_{ID}$ (128 bits, 16 bytes).

**Figure 2:**
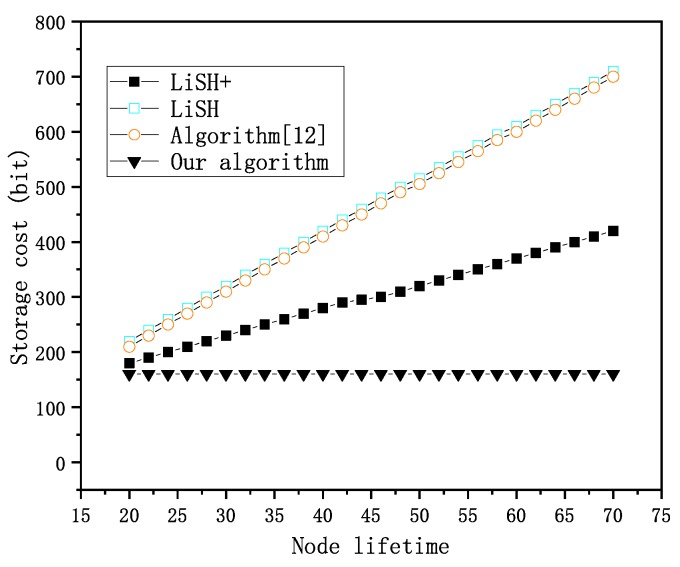
Storage cost.

The key distribution scheme proposed in [23], as well as the LISH and Lish+ algorithms, their node storage cost is related to the lifetime of the node, and its correlation is shown in Figure 2. Figure 2 shows the comparison of the security key establishment mechanism based on high-order polynomial and the communication cost of the above key distribution schemes.

In Figure 2, the security key establishment mechanism based on high-order polynomial requires the smallest storage space, and it does not increase with the node’s lifetime. Therefore, the key establishment mechanism based on high-order polynomial is superior to the other schemes on storage cost.

### 4.2. Communication Cost

Using the security key establishment mechanism based on polynomial coefficients proposed in this paper, in a time slot (i.e., a key update period), the information about the key update that needs to be transmitted in the communication channel includes: a key update polynomial coefficients, the list of attacked components, and the list of random numbers. The number of the key update polynomial coefficients is related to the number of normal components *m* in the cluster. In general, the number of key update polynomial coefficient is m+2. The list of attacked components lists all the ID of attacked components, and the communication cost about it is related to the number of attacked components *p*. The random number list contains some random numbers used to construct the key update polynomial; the number of the random number *x* is related to the number of normal components *m* and the number of attacked components *p* in the cluster.

**Figure 3:**
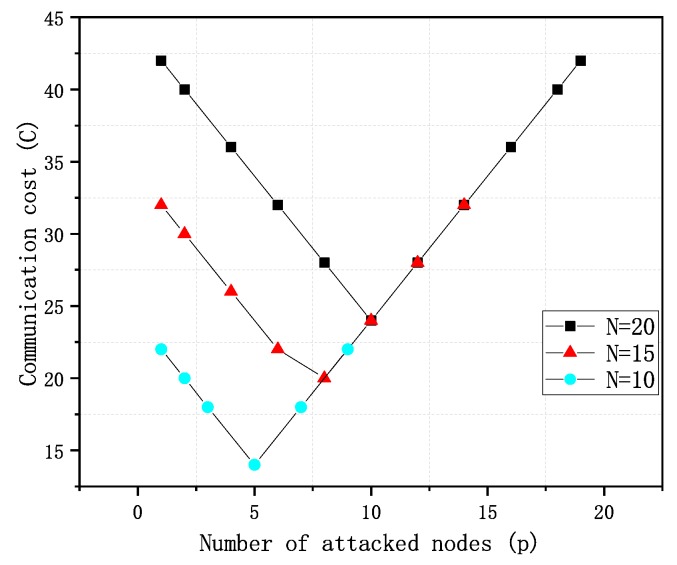
Communication cost.

Therefore, when the number of normal communication components is greater than or equal to the number of attacked components in the cluster, that is, m>=p, the communication cost is C=m+2+p+x=2(m+2). When the number of normal communication components is less than the number of attacked components in the cluster, that is, m<p, the communication cost is C=2(p+2). In general, there are several to a dozen components that need to communicate with each other, thus there are not many components communicating with each other within one cluster. Figure 3 shows the relationship between communication cost and the number of components being attacked when the total number of components in the cluster is 10, 15, and 20. Figure 3 shows that the communication cost is the lowest when the number of normal components and the number of attacked components in the cluster are equal. When there are more normal components in the cluster are attacked components, the communication cost is negatively correlated with the number of attacked components. When there are fewer components not being attacked than components being attacked, the communication cost is positively correlated with the number of components being attacked.

## 5. Conclusions

This paper focuses on the generation and distribution of communication keys between components in robots. A security key establishment mechanism based on high-order polynomial is proposed, and key generation is performed in a cloud-based key center. Prior to the key update, a key update instruction, which includes the coefficients of a group polynomials and the attacked components list, are broadcast to the components in a cluster. The coefficients ensure that the components which are in the secure state can correctly calculate the key, while the components which are being attacked cannot obtain the key. The key establishment mechanism proposed in this paper guarantees the security of the key. At the same time, the communication components only need to do simple calculation to get the key and the storage cost is small, which is more suitable for the situation where the resources are limited in some of the robots. Compared with the Lish and Lish+ algorithms, our mechanism allows robot components to dynamically join and leave the network, which is more suitable for multi-robot systems.

## Figures and Tables

**Table 1:** The Comparison with Existing Solutions.

Algorithm	Storage Cost	Communication Cost	Revocation	Collusion Resistance	Robustness
Algorithm [22]	2logq	1logq	Limited	×	×
Algorithm [23]	$(2s_{2}-2s_{1}+4)logq$	(h+(1+e)/2)logq	✓	Part	×
LiSH	$(2s_{2}-2s_{1}+6)logq$	(h+l+1)logq	✓	✓	✓
LiSH+	$(h+s_{2}-s_{1}+6)logq$	(h+l+1)logq	✓Dynamic	✓	✓
our mechanism	160 bits	2(m+2)/2(p+2)	✓Dynamic	✓	✓

* *e* indicates the number of communication sessions, *h* indicates the number of polynomials, *q* is a large prime number greater than the number of components in the network, *l* is the size of the key buffer, *m* indicates the number of normal components, *p* indicates the number of abnormal components, and $\left[s_{1},s_{2}\right]$ indicates the lifetime of a user; ** (t+(1+m)/2)logq is the average communication cost of the Du scheme, and the cost of the algorithm in session j is calculated as (t+j)logq.
